# Recording Spikes Activity in Cultured Hippocampal Neurons Using Flexible or Transparent Graphene Transistors

**DOI:** 10.3389/fnins.2017.00466

**Published:** 2017-08-28

**Authors:** Farida Veliev, Zheng Han, Dipankar Kalita, Anne Briançon-Marjollet, Vincent Bouchiat, Cécile Delacour

**Affiliations:** ^1^Institut Néel, Centre National de la Recherche Scientifique and Université Grenoble Alpes Grenoble, France; ^2^Grenoble Alpes, HP2 Laboratory, Institut National de la Santé et de la Recherche Médicale U1042 Grenoble, France

**Keywords:** graphene, transistor array, hippocampal neurons, bioelectronics, neural interfaces, electrophysiology, neuroelectronics

## Abstract

The emergence of nanoelectronics applied to neural interfaces has started few decades ago, and aims to provide new tools for replacing or restoring disabled functions of the nervous systems as well as further understanding the evolution of such complex organization. As the same time, graphene and other 2D materials have offered new possibilities for integrating micro and nano-devices on flexible, transparent, and biocompatible substrates, promising for bio and neuro-electronics. In addition to many bio-suitable features of graphene interface, such as, chemical inertness and anti-corrosive properties, its optical transparency enables multimodal approach of neuronal based systems, the electrical layer being compatible with additional microfluidics and optical manipulation ports. The convergence of these fields will provide a next generation of neural interfaces for the reliable detection of single spike and record with high fidelity activity patterns of neural networks. Here, we report on the fabrication of graphene field effect transistors (G-FETs) on various substrates (silicon, sapphire, glass coverslips, and polyimide deposited onto *Si/SiO*_*2*_ substrates), exhibiting high sensitivity (4 mS/V, close to the Dirac point at V_LG_ < V_D_) and low noise level (10^−22^ A^2^/Hz, at V_LG_ = 0 V). We demonstrate the *in vitro* detection of the spontaneous activity of hippocampal neurons *in-situ*-grown on top of the graphene sensors during several weeks in a millimeter size PDMS fluidics chamber (8 mm wide). These results provide an advance toward the realization of biocompatible devices for reliable and high spatio-temporal sensing of neuronal activity for both *in vitro* and *in vivo* applications.

## Introduction

The current non-invasive technologies (for instance functional MRI, EEG) enable the real-time monitoring of the whole brain activity. Those techniques have already provided impressive mapping of neuronal architectures *in-situ* (Zupanc, 2017). However, these far-field approaches integrate signals of a large population of cells (volume around mm^3^) and are far below single-cell resolution. For neuro-rehabilitation process, one needs to stimulate, detect, and track the activity of specific single neurons, as shown for restoring accurate motor motion (Hochberg et al., 2012; van den Brand et al., 2012) sensory feedbacks (Raspopovic et al., 2014) or vision (Hornig et al., 2005). To get a closer access to single cells and sub-cellular nanoscale events, optical techniques could be used for instance to manipulate ion channel activity (Szobota and Isacoff, 2010), to follow sub-threshold electrical signals along neuronal arborization (Zecevic, 1996; Tanese et al., 2017) or to track neurotransmitters release (Nicovich et al., 2017). Also, electronics devices can provide quantitative information and are still required for long lasting recordings or when interfacing unaltered (genetically or stained) cells.

The current devices which are widely used since the 1980's for detecting the electrical activity of brain cells consist of microelectrodes arrays (MEA) (Thomas et al., 1972; Spira and Hai, 2013). They form capacitive systems with the surrounding cells and tissues, which can sense small variations of local field potential or even spikes, and can also stimulate the cells electrical activity thanks to their high interfacial impedance. Already used a few centuries ago by Galvani for stimulating frogs muscles, the reduction of the electrodes size has started with the emergence of microelectronics. Today, they allow accurate mapping of neural network activity with single cell resolution (Huys et al., 2012; Bakkum et al., 2013; Müller et al., 2015). The fabrication process, the electronics, and the spike sorting analysis are now well advanced and the technology is commercially available. Also, several materials have been tested so far, such as, diamond (Piret et al., 2015), platinium (Oka et al., 1999), gold (Brüggemann et al., 2011), carbon nanotubes (Keefer et al., 2008; Suzuki et al., 2013), and vertical nanowires (Robinson et al., 2012). However, there is still an interest in new devices and materials. The main drawback of microelectrodes is the size issue. The electrode probing area cannot be reduced while keeping high signal-to-noise ratio. Indeed, the detection being based on charges injection, the current response is inversely proportional to the electrode/electrolyte impedance. Therefore, a reduction of the electrode surface results in an increased impedance and reduced signal-to-noise ratio.

In order to detect low transient changes of extracellular potential associated to neural spikes (~10 μV), the electrodes areas *S* need to be typically around 2–50 μm in diameter and thus are much larger than single neurons (around 1 and 10 μm for neurite and soma). Today, even the smallest MEAs cannot provide access to single neurites, synapses, and ion channels (Spira and Hai, 2013). However, this activity at or below the single cell limit is involved in numerous brain processes such as, plasticity (Ribrault et al., 2011) and channelopathies (Ashcroft, 2006) or neurodegenerative diseases. Also, monitoring the propagation of single spikes along neural architectures could be crucial for cells biology, pharmaceutics, and medical healthcare. For that purpose, higher spatio-temporal resolutions are required as well as reliable and long lasting devices.

Since few years, field effect transistors (FETs) have attracted particular attention. Based on a modulation of the charge carrier density as function of the surrounding electrical field, the FET sensitivity is proportional to the width-to-length aspect ratio *W/L* of the transistor channel instead of its surface: (RSze et al., 1981)

$$\frac{\partial I_{DS}/\partial V_{LG}}{V_{DS}}=\frac{g_{m}}{V_{DS}}=\mu .C_{EDL}\frac{W}{L}$$

with *g*_*m*_ the FET transconductance, *I*_*DS*_ *and V*_*DS*_ the drain source current and voltage, *V*_*LG*_ the gate voltage, μ the charge carriers mobility, *C*_*EDL*_ the interfacial capacitance. Thus, the device size can in principle be downscaled way below the single cell limit while still keeping significant sensitivity, allowing spikes tracking along neurites and neural networks (Hutzler et al., 2006; Patolsky et al., 2006) and the detection of microscopic events such as, neurotransmitters release (Wang et al., 2007), DNA bases (Kim et al., 2004), or proteins recognition (Chen et al., 2011).

Meanwhile, graphene has emerged as promising 2D platform for bio- and neuro-electronics (Schmidt, 2012; Kostarelos et al., 2014) regarding the high chemical stability and biocompatibility of the *sp*^2^-hybridized carbon lattice. Moreover, the higher charge carriers motilities and the absence of top gate oxide have allowed to overcome the threshold performance of the semiconductors technology with higher transconductance and devices sensitivity (S = 4.23 mS/V for G-FET vs. 0.2 mS/V for silicon FET; Hess et al., 2011a).

Large sheets of polycrystalline monolayered graphene can be grown by chemical vapor deposition on copper foils and further transferred on a wide range of substrates at the manufacturing level (Bae et al., 2010) allowing large-scale integration of the devices on transparent, soft, and flexible substrates suitable for real time optical stimulation or imaging of the interfaced cells. Also, the mechanical properties of graphene allow the implementation of stretchable—and still highly conductive—electronics for matching the non-planar geometry of cells and keeping an intimate contact. Together with its flexibility, its possible implementation on soft substrates could reduce the inflammatory response of the body which currently prevents reliable and long lasting neural interfaces *in vivo* (Kuzum et al., 2014; Jeong et al., 2015).

Moreover, the positive impact of graphene on cells regrowth and maturation have been shown in numerous studies including primary neurons (Bendali et al., 2013; Veliev et al., 2016) and stem cells (Park et al., 2011) without altering their electrical activity (Fabbro et al., 2016). The cytocompatibility is certainly one of the main advantages of graphene in comparison with other 2D materials. The high adhesion on pristine graphene (without any additional adhesive coating) provides also high sealing with neurons, a feature which is a key parameter for extracellular measurement, while the fast regrowth of neurites could promote the regeneration of the damaged neural network when interfacing native tissues or dissociated cells in culture. Also, the small amount of implanted materials is an advantage to reduce the toxicity risks. Indeed, several studies have shown that macrophages degrade efficiently graphene flakes when introduced in several organs of the body (Bianco, 2013; Girish et al., 2013).

Since the past few years, graphene field effect transistors (G-FETs) have been able to provide versatile detectors that enabled to sense low pH change (Ohno et al., 2009) DNA translocation (Xu et al., 2017) cancer cells (Feng et al., 2011), or bacteria (Mannoor et al., 2012). Also, G-FETs were able to detect single spike evoked in electrogenic cells line, such as, cardiomyocytes (Cohen-Karni et al., 2010) or HEK/PC12 cells (Hess et al., 2011b) and more recently ion channel activity (Veliev et al., 2017) and slow potential waves resulting from synchronous activity of a large population of neurons have been recorded by electrocorticograms performed on living rats (Blaschke et al., 2017). Compared to previous results, one has to note that neurons are much smaller, fragile, and less accessible. However, these results hint the possibility to record single spike from single neurons with an implantable graphene based bioelectronics.

Here, we report on the fabrication of G-FETs arrays on transparent and flexible substrates (sapphire, glass, coverslip, and polyimide substrates, still compared with conventional silicon on insulator substrates) and their ability to record the spontaneous activity of primary hippocampal neurons cultured *in-situ*, on the chip, in a microfluidic chamber, during 21 days until the maturation of the network was complete.

## Results and discussions

Graphene monolayers are grown by chemical vapor deposition on 25 μm thick copper foils (99.8% purity, Alfa-Aesar) by decomposition of methane gas *CH*_*4*_ at 1,000°C in diluted hydrogen atmosphere as previously described (Han et al., 2014). After cleaning in acetone, the cooper foils is annealed in diluted *H*_*2*_ atmosphere (dilution in *Ar* at 10%) at 1,000°C for 2 h to reduce the native copper oxide and enlarge the copper grains. Then, pulses of methane *CH*_*4*_ (2 sccm 10 s, then 60 s off) are injected as a carbon source, instead of using continuous flow of methane, to prevent the aggregation of carbon at the nucleation centers and avoid the formation of multi-layers patches. This pulse growth enables to reach higher carriers mobilities values up to 6,700 cm^2^/V/s (Han, 2013).

**Figure 1:**
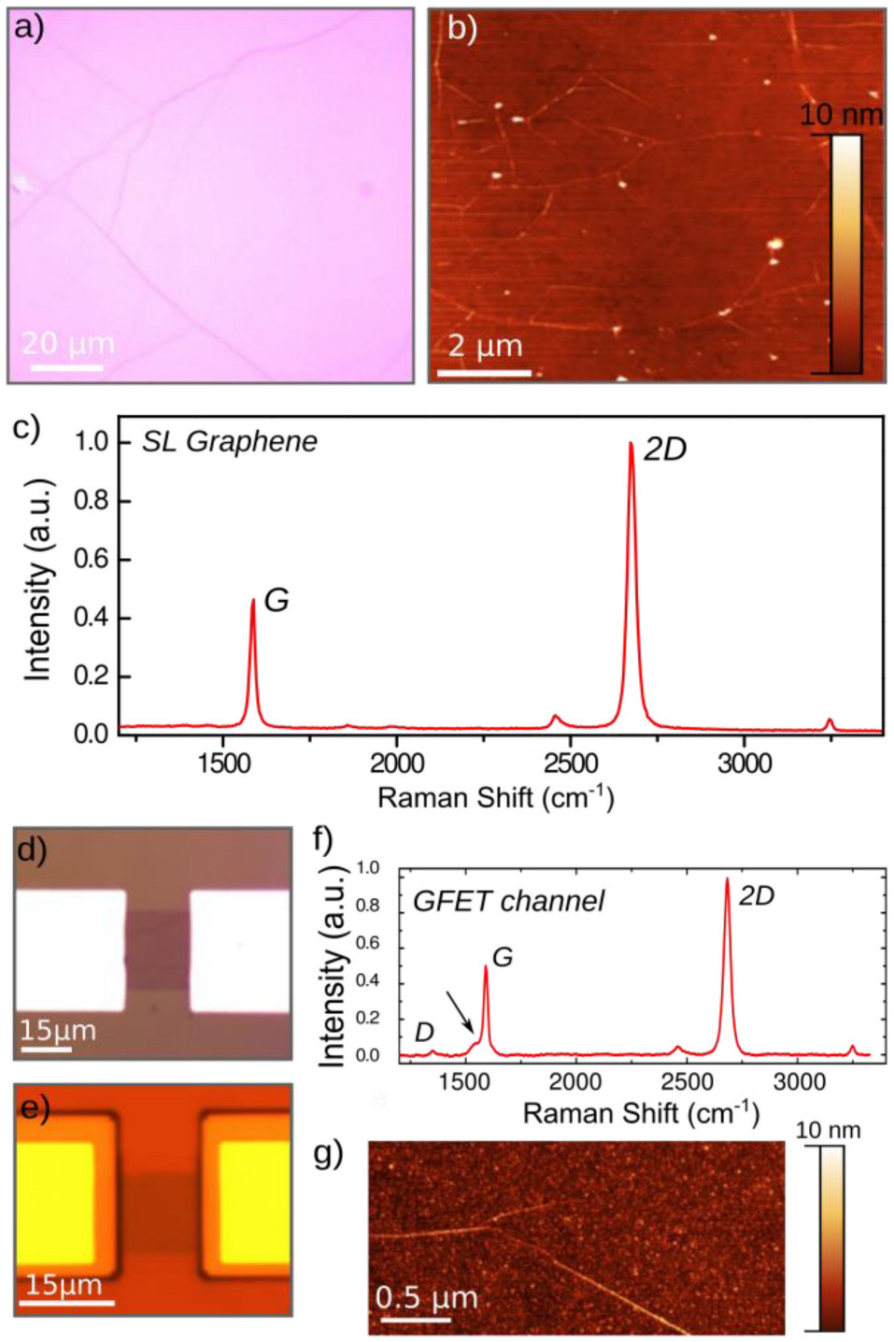
Surface and structural characterizations of the CVD-grown graphene monolayers. **(a)** Optical micrograph of a typical graphene monolayer grown by pulsed CVD, after transfer on oxidized silicon (285 nm SiO_2_). **(b)** Atomic force micrograph of the graphene surface after transfer and annealing at 300°C for 3 h. **(c)** Raman spectroscopy underlying the characteristic Raman peaks of graphene monolayers (detailed in the text). Optical images of the graphene channel (20 × 15 μm^2^) before **(d)** and after **(e)** passivation. **(f)** Raman spectrum of the active graphene FET channel of the final device after contact passivation by photoresist. **(g)** Atomic force micrograph of the graphene FET channel after passivation.

Pieces of Cu foil of about 4 × 4 mm^2^ with graphene monolayers on top, are then transferred on the substrates—Si/SiO_*2*_, sapphire, glass coverslip (Marienfeld), and polyimide (deposited on *Si/SiO*_*2*_ substrates)—by wet transfer technique. Briefly, a plastic carrier (spin-coated PMMA film) is deposited on top of the graphene layer, then the copper foils is etched in solution containing specific Cu etchant [*(NH*_*4*_*)*_*2*_*S*_*2*_*O*_*8*_ at 0.1 g/ml]. Once the Cu foil is completely dissolved, the bilayer (Graphene-PMMA) is rinsed three times in deionized (DI) water to remove the etchant and is then wet-transferred onto the host substrate. The PMMA resist is removed in acetone (overnight bath), then the sample is rinsed and annealed at 300°C for 3 h in diluted hydrogen atmosphere (H_2_/Ar, mbar) to remove PMMA residues. Optical and atomic force micrographs (Figures 1a,b, respectively) show the overall continuity and homogeneity of the transferred graphene monolayer and the quasi absence of PMMA residues. Micro-Raman spectra assess for the crystalline quality of the transferred graphene monolayer. Each trace represents the averaged value of several measurements performed at different points around the channel. Figure 1c shows the two main Raman lines of graphene, the G-mode (1,580 cm^−1^), and 2D-mode (2,700 cm^−1^). Their widths *w*_2D_ = *24 cm*^–*1*^ and intensity ratio *I*_2*D*_*/I*_*G*_ = *0.3* are as expected for single graphene layer (Ferrari and Basko, 2013). The absence of the D-peak (expected around *1,350 cm*^–*1*^) confirms the high quality of the monolayer.

The transistors arrays are micro-fabricated by first etching the graphene in stripes that will act as transistor channels. This is done by patterning channels with a photoresist mask and etching of the unprotected parts using oxygen plasma. Contact leads are then deposited using a photoresist mask and evaporation in vacuum of metal thin films (Ti/Pt/Au or Pd). Pd was used as the metal contact on sapphire substrates, while Ti/Pt/Au was rather used on PID and glass coverslips to enhance the adhesion of the metal onto the substrates. For SiO2 substrates, we have used both Pd and Ti/Pt/Au (for comparison with the sapphire, and the glass or PID substrates, respectively). The contact resistance is qualitatively estimated by measuring the two-point resistance of G-FETs with different widths, being around *R*_*C*_ = *0.3 k*Ω, per contact, while the square resistance *R*_□_ of the graphene sheet is about *R*_□_ = *0.65* ± *0.05 k*Ω*/*_□_ (Veliev et al., 2017). Finally, the metallic contact leads are insulated using biocompatible polymers, either polyimide (Fujifilm, photosensitive PID) or the negative photoresist SU8-2000, and are annealed during 2 h at 200°C in N2 atmosphere, and 30 min at 150°C, respectively. No significant difference was observed on the GFETs performances. However, the SU8 provides a lower fluorescent background noise for the immuno-fluorescent stainings used to locate the cells after the recordings. Smallest G-FETs arrays with channel width and length of W × L = 20 × 15 μm^2^ were realized (Figure 1d). The effective gate length (graphene area exposed to the liquid gate) is reduced to 10 μm after passivation of the metal contact leads, regarding the overlaps between the graphene channel and the passivation layer (Figure 1e). Optical micrographs of the graphene FET channel show the absence of large contaminant after deposition (Figure 1d) and passivation (Figure 1e) of the metal contacts. For some devices, a common ground electrodes were designed to increase the number of GFETs (80 per chips), as shown in **Figure 4** for instance.

To detect a possible degradation of the crystalline quality of graphene by the FET fabrication process, Raman is performed after process (Figure 1f). The integral intensity ratio *I*_*G*_ */I*_2*D*_ = *0.33* of the G and 2D mode peaks, as well as the width of the 2D peak ω_2*D*_ = *31 cm*^–*1*^ and the low intensity of the defect-induced D-peak, reveal the high quality of graphene even after the FETs fabrication. A new peak appears on the right shoulder of the G-peak however, which could indicate the presence of resist residuals. Atomic force micrographs reveal indeed a slight (nanometer scale) contamination of the surface (Figure 1g).

**Figure 2:**
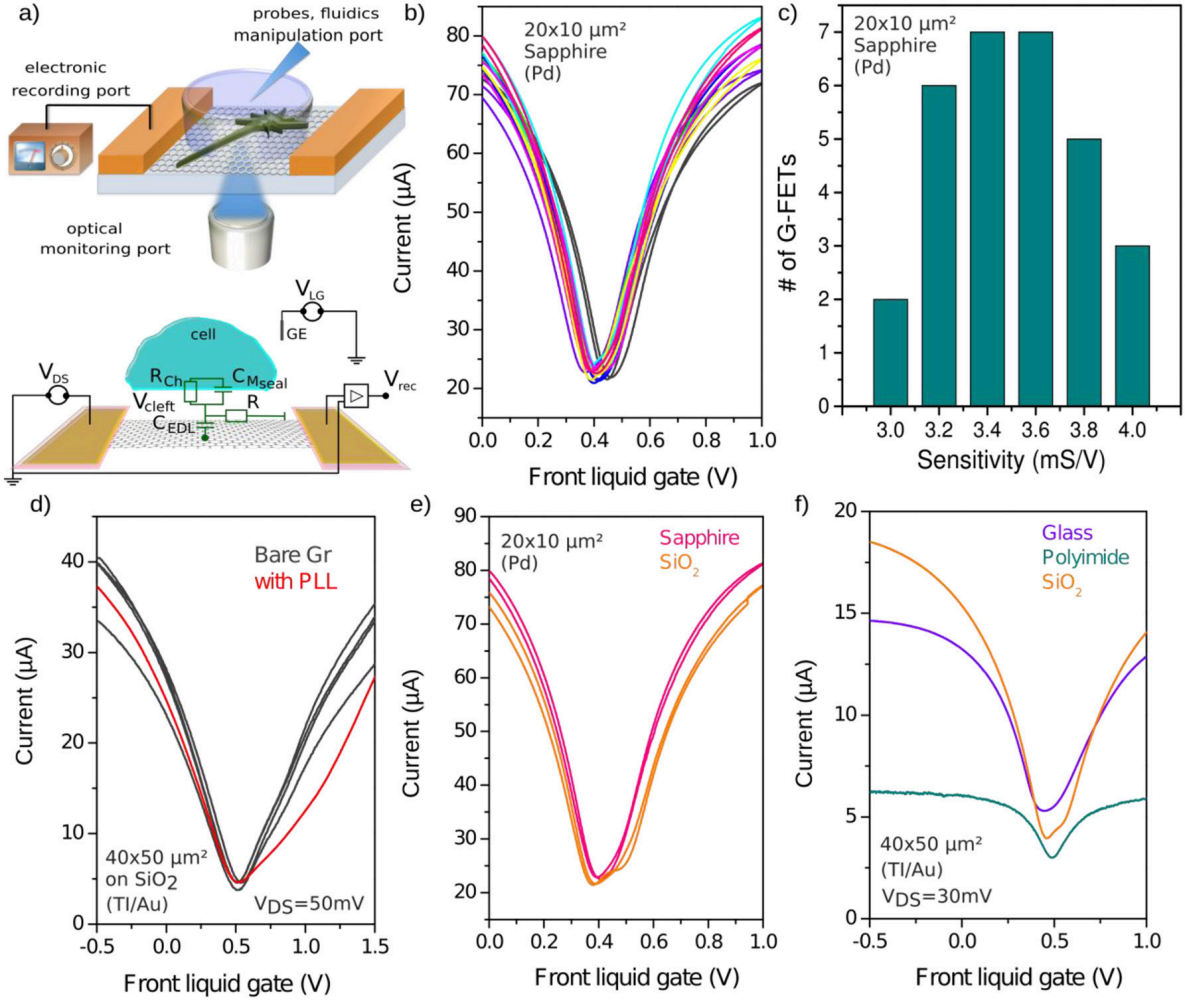
Performance of G-FETs arrays on several substrate. **(a)** Schematic view of an inverted microscope setup using transparent graphene electronics for simultaneous electrical and optical recording, cell control and fluidic manipulation. The electrical equivalent circuit of the graphene digital interface is described below. **(b)** Field effect characteristics of liquid-gated field effect measured on 10 devices showing reproducible behavior and narrow distribution of parameters. The measurements were performed on 20 × 10 μm^2^ G-FETs fabricated on sapphire at V_DS_ = 75 mV with Pd/Au metallic contacts. **(c)** Sensitivity distribution of 30 identical FETs on the same chip than **(b)**. **(d)** Field effect characteristics of liquid-gated G-FETs (40 × 50 μm^2^) on SiO_2_ before and after covering with poly-L-lysine (PLL). **(e)** Comparison of liquid-gated G-FETs (20 × 10 μm^2^) fabricated on SiO_2_/Si and on sapphire substrates (V_DS_ = 75 mV, Pd metallic contacts) and **(f)** the G-FETs (40 × 50 μm^2^) fabricated on SiO_2_/Si, Glass and PID substrates (V_DS_ = 30 mV, Ti/Pt/Au metallic contacts). All measurements were performed in cell culture medium using Pt reference electrode.

The sensitivity of the fabricated G-FETs is measured with a liquid top gate, with either cell culture medium or PBS solution, and biased with a Pt or Ag/AgCl reference electrodes. Either Pt or Ag/AgCl reference electrode could be used as a gate electrode, as both gate electrodes showed identical transconductance. Due to the high capacitance of the electrical double layer at the graphene/liquid interface (*C*_*EDL*_ ~*2* μ*F/cm*^*2*^), a low variation of the liquid gate potential could induce a high modulation of the drain-source current *I*_*DS*_ through the transistor channel. By increasing the gate potential, the transistor operation mode varies from hole to electron conduction regime by passing the conductance minimum at the charge neutrality point (Dirac point), typically around *V*_*CNP*_ ~ *0.4 V*, depending on the substrates and the used reference electrodes. Figure 2a depicts the principle of the neuron-covered liquid-gated FET (top) and the electrical setup (bottom), while Figure 2b shows the reproducible field effect responses of the G-FETs gated with the liquid potential, showing the reliability of the fabrication process per chip.

The sensitivity of the G-FET is proportional to the G-FET transconductance—defined as the differential conductance *g*_*m*_ = ∂_*IDS*_*/*∂_*VLG*_—normalized by the bias drain-source voltage such as, *S* = *g*_*m*_ */V*_*DS*_. The mean value of the G-FETs sensitivity is around S = 3.5 mS/V, and maximal values reaches 4 mS/V for the 20 × 10 μm^2^ G-FETs on sapphire substrate (Figure 2c). These values are comparable with the highest reported G-FET performance for CVD grown graphene(Hess et al., 2011a).

Coating the sample with a capping layer that promotes cell adhesion is widely used to ensure high attachment and neurites regrowth onto the samples, especially for primary neurons which require weeks of culture for establishing a mature (electrically connected) network. Synthetic polymers such as, poly-L-lysin are generally used to bond the neurons membranes by taking advantage of electrostatic interactions. However, as these polymers are intercalated between graphene and neurons, they form a charged layer that affects carriers mobility within the graphene channel and thus lead to decrease signal-to-noise ratio by partially screening the neuron gating signal. Indeed, after functionalization, the conductance Vs Gate curve shows a slightly broadened dip and exhibits two local minima, suggesting the presence of fluctuations of doping levels in graphene (Figure 2d). However, the transconductance on both hole and electron sides is barely affected by the coating, as expected for a non-covalent PLL/Graphene interactions (Wang et al., 2013) and thus the G-FET sensitivity remains the same with and without the PLL coating, at least in the highest sensitive operating points.

**Figure 3:**
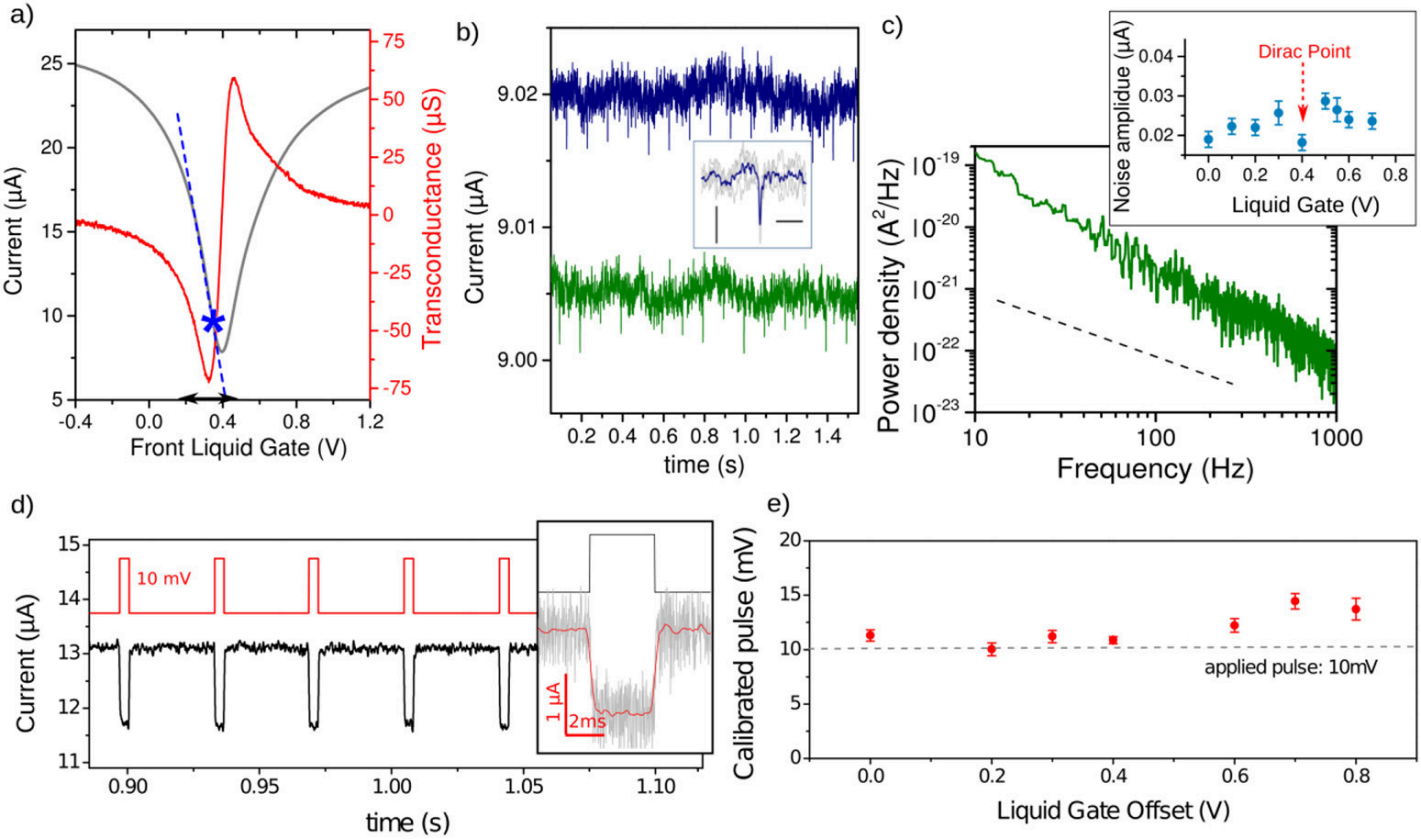
Noise and stability of the G-FETs for potential pulse detection. **(a)** Field effect and transconductance of the G-FETs measured in the cell culture medium. Dashed blue line indicates the linear operation regime of the G-FET with the maximal sensitivity. The black arrows highlight the gate voltage range of the maximal sensitivity around V_LG_ = 0.3 V. The blue star indicates the operating point for the pulses detection shown in **(b)**. **(b)** Detection of 100 μV (blue) and 75 μV (green) potential pulses (applied to the cell culture medium through a Pt-electrode) by G-FETs biased at V_*DS*_ = 50 mV and V_LG_ = 0.32 V. Inset: zoomed of the superimposed G-FETs responses. Scale bars are 2.5 nA and 10 ms **(c)** Power spectral density of the current noise in liquid gated G-FETs (V_LG_ = 0 V). A 1/f dependence on the frequency is observed (dashed line). Inset shows the noise amplitude as function of the liquid gate voltage. **(d)** G-FET response (black line) to square shaped potential pulses (red line) applied in cell culture medium. Inset: zoom of a pulse detected by G-FET (blackline), revealing a very fast response (~0.5 ms) to the applied square pulse signal (redline). Gray line is the unfiltered signal. **(e)** Calibrated pulse height from GFET transconductance **(a)** of the potential pulse V_*P*_ = 10 mV showing that the calibrated pulse and the applied pulse are in very good agreement.

A main advantage of graphene and 2D materials is the possibility to integrate them on transparent and flexible substrates allowing a see-through operation compatible with optical microscope or microfibers *in vivo* (see Figure 2a). Figures 2e,f compare the transconductance (*I*_*DS*_*-V*_*LG*_ curves) of the G-FETs supported on diverse materials such as, glass coverslips, sapphire, and polyimide in comparison with conventional *Si/SiO*_*2*_ substrates. While the electrical characteristics of G-FETs fabricated on glass coverslip and *Si/SiO*_*2*_ are almost identical—the square-normalized sensitivity *S* = *S*_□_ × *L/W* being 1.2 mS.V^−1^._□_ and 1.36 mS. V^−1^._□_ for glass coverslip and *Si/SiO*_*2*_, respectively—the devices realized on polyimide exhibit higher contact resistance and a slightly shifted Dirac point toward higher doping level (with *S*_□_ = 0.8 mS.V^−1^._□_). Nevertheless, the extracted transconductance values are almost similar for all substrates, indicating that one performance limitation of G-FETs fabricated on glass or soft PID substrates could be the higher contact resistance. The devices fabricated on sapphire show highest sensitivity value (*S*_□_ = 8 mS.V^−1^._□_) which could result from a lower density of charges trapped at the interface with the substrate. This performance is one of the highest reported for CVD grown grapheme (Hess et al., 2011a). We tested the ability of the GFETs to detect potential pulses with shape and amplitude similar to the expected extracellular neuronal spike. At the operation point (the highest operating regime, underlined with the blue star Figure 3a), GFETs enable the detection of short pulses (1 ms) of low amplitude (75 μV) applied to the cell culture medium through a Pt-electrode (Figure 3b). The power spectral noise density of the G-FETs in two-points measurement configuration shows a dependence in frequency according to 1/f law for the low frequency regime with a noise level around 10^−22^ A^2^/Hz at 1 kHz (Figure 3c). This low noise level is expected for graphene in comparison to silicon, and could be in part explained by the absence of top gate oxide which acts as an additional source of noise in semiconductors devices (Balandin, 2013). This density or mobility fluctuations within the FET channel could result either from charge traps at the interface with the substrates, or from defects at the edges of the graphene channels that can contribute to the Generation-Recombinaison G-R noise (Rumyantsev et al., 2010).

The G-FETs provide a fast response time (at least faster than spike duration), with a rise time of τ ~ *0.5 ms* (Figure 3d) and reliable detection of potential spikes applied in the liquid gate (Figure 3e), the minimal amplitude being *V*_*P*_ = *75* μ*V* with a signal-to-noise ratio around S/N ~2.5 (Figure 3b), which should allow the extracellular detection of neuronal spikes. The detection performance may vary depending on the cell/device coupling area and fluctuation around the operating point, which could arise from many processes when interfacing cells, such as, molecules adsorption or local pH changes. This could be overcome by low contact resistance which increases the range of the linear and highest sensitive regime (underlined with the arrow Figure 3a).

**Figure 4:**
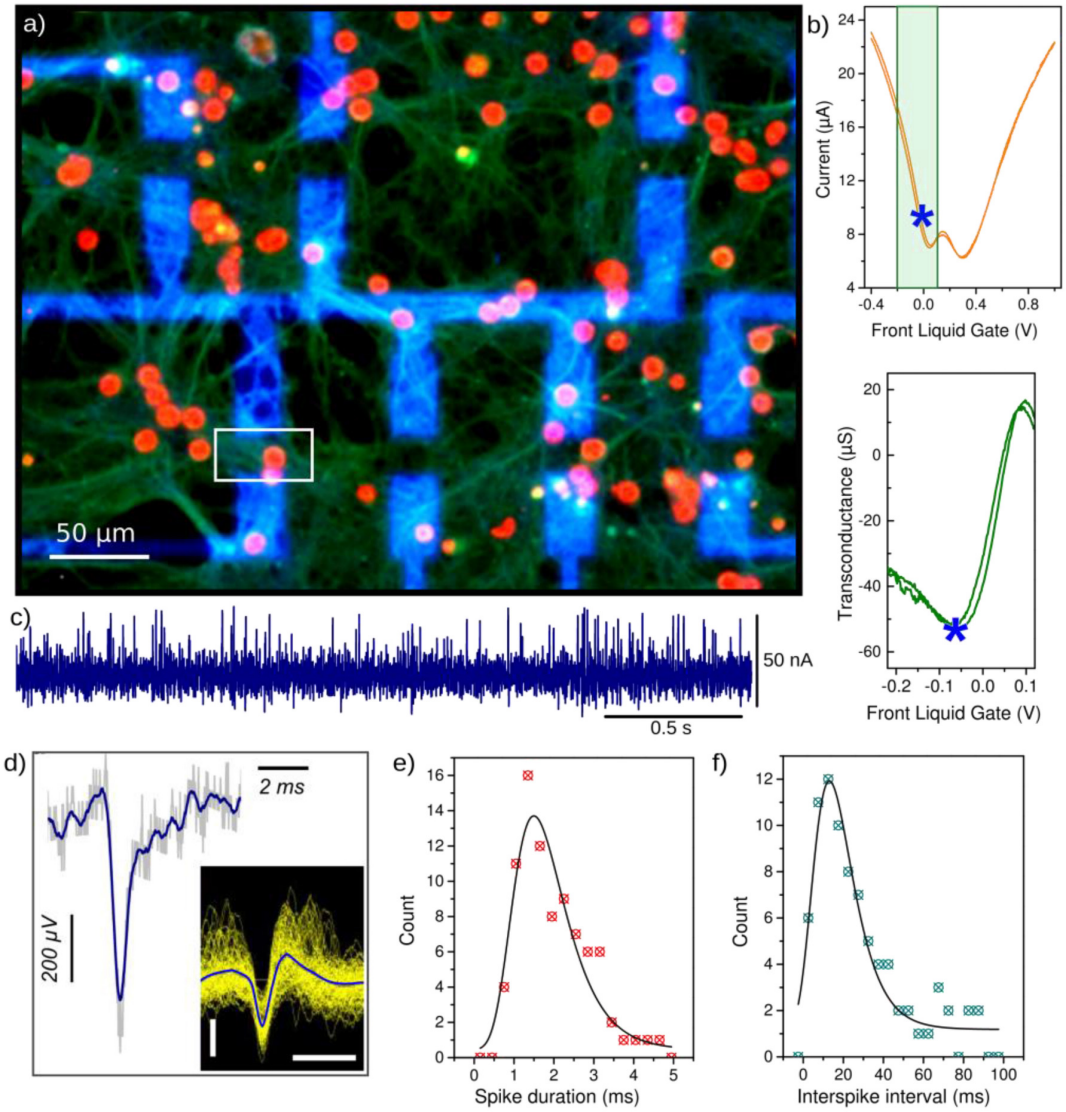
Primary hippocampal neurons interfaced with G-FETs (20 × 10 μm^2^). **(a)** Immuno-fluorescence micrographs of neurons on the G-FETs after 21 days of culture, stained with DAPI (red) and anti-Synapsin (green) to label the soma and the synaptic vesicles along the neuritis, respectively. The metal contact leads appear in blue and connect the graphene FET channel (the position of one G-FET is underlined with the white square for example). **(b)** G-FET current vs. liquid gate voltage, set using a Ag/AgCl reference electrode immersed into the cell culture medium (top) and corresponding transconductance curve (bottom) in the highest sensitive regime (underlined by the green square on the top curve). The blue star indicates the transistor working point with the highest sensitivity in the hole conduction regime. **(c)** Recording of neuronal activity by monitoring the *I*_SD_ current of the G-FET underlined with a white square in **(a)**, at the transistor working point indicated by a blue star in **(b)**. The measurements were performed in cell culture medium (V_SD_ = 100 mV). **(d)** The calibrated extracellular potential obtained from a single current spike, obtained from **(c)** and the transconductance value at the operating point (detailed in the text). The inset shows the superposition of all detected spikes. The scale bars are 200 μV and 2 ms. **(e)** Histograms showing the distribution of the spike duration and **(f)** distribution of the interspike interval (right).

Primary neurons were cultured (Veliev et al., 2016) above the sensors during 21 days until the maturation of the network was complete. Briefly, the hippocampi are extracted from E16 mouse embryo. Neurons are mechanically dissociated and seeded (6 10^4^ cell/cm^2^) onto the sterilized samples in attachment promoting medium (MEM supplemented with 10% fetal bovin serum). Samples were previously coated with poly-L-lysin (at 100 μg/ml over night, then rinsed) to enhance the adhesion of neurons over the surface. After 2 h, the medium is replaced by a serum free and glial conditioned neurobasal supplemented with AraC (cytosine arabinoside, at 1 μM) to prevent the proliferation of glial cells which would form a barrier between the sensors and the neurons. Neurons can be observed on the transparent sapphire or glass samples with conventional optical microscope, and be compared with control glass coverslips (without devices) during the culture time. After the measurements, neurons are fixed in 4% paraformaldehyde and immuno-histologies assess precisely the cells position and shape above the sensors, by labeling the soma, the synapses and the micrometer size neurites and axons, with DAPI (4′,6-diamidino-2-phenylindole, at 1 μl/ml), and anti-Synapsin (2 μg/ml), anti-YL1/2 (1 μg/ml), and anti-Tau (2 μg/ml) primary antibodies, respectively (Figure 4a).

The neuronal activity is monitored after 19–21 days of culture, in CO_2_ and temperature regulated atmosphere, using a needle probe station interfaced with FPGA electronics. The liquid gate voltage *V*_*LG*_ is applied to a Pt or Ag/AgCl electrodes immersed into the solution. A constant DC drain-source bias voltage *V*_*DS*_ is applied to the G-FET and the drain-source current through the transistor channel *I*_*SD*_ is monitored using commercial and home made current amplifiers. To decrease the input noise, voltage dividers and low pass filters are used. The output signal is filtered using a low pass filter with a cut-off frequency *f*_*c*_ = *5 kHz*.

Immuno-fluorescent *IF* micrograph of neurons cultured on the G-FETs arrays is shown in Figure 4a, and the electrical properties of the interfaced G-FETs in Figure 4b. The recorded *I*_*SD*_ traces exhibit short current pulses, which duration, amplitude and polarity are as expected for neuronal signals (Figure 4c). Using the transconductance *g*_*m*_ at the set transistor operating point (underlined with the stars in Figure 4b), the extracellular potential can be estimated to *V*_*extra*_ = *I*_*SD*_*g*_*m*_. The calibrated extracellular potential is shown in Figure 4d. The amplitude is around 570 ± 80 μV, and the spike duration τ = 1.5 ± 0.1 ms (Figure 4e). The polarity and the shape of the detected signals correspond to the expected extracellular potential spike generated by the opening of sodium channels and influx of positively charged Na^+^ ions into the cell resulting in a negative potential change in the cleft and a positive variation of *I*_*DS*_. The signal amplitude seems high compared to values reported using MEAs (Spira and Hai, 2013) however for SiNW-FETs, extracellular action potentials with amplitude up to several mV were reported (Voelker and Fromherz, 2005). The interspike interval (ISI) Δt = 13 ± 0.8 ms (Figure 4f) is in good agreement with values reported for *in vivo* conditions (Chorev and Brecht, 2012) however slightly shorter than expected for *in vitro* neuronal cultures. But since the neuron density is high, it could be possible that the device (underlined with a white square Figure 4a) is recording action potentials originating from more than one neuron.

**Figure 5:**
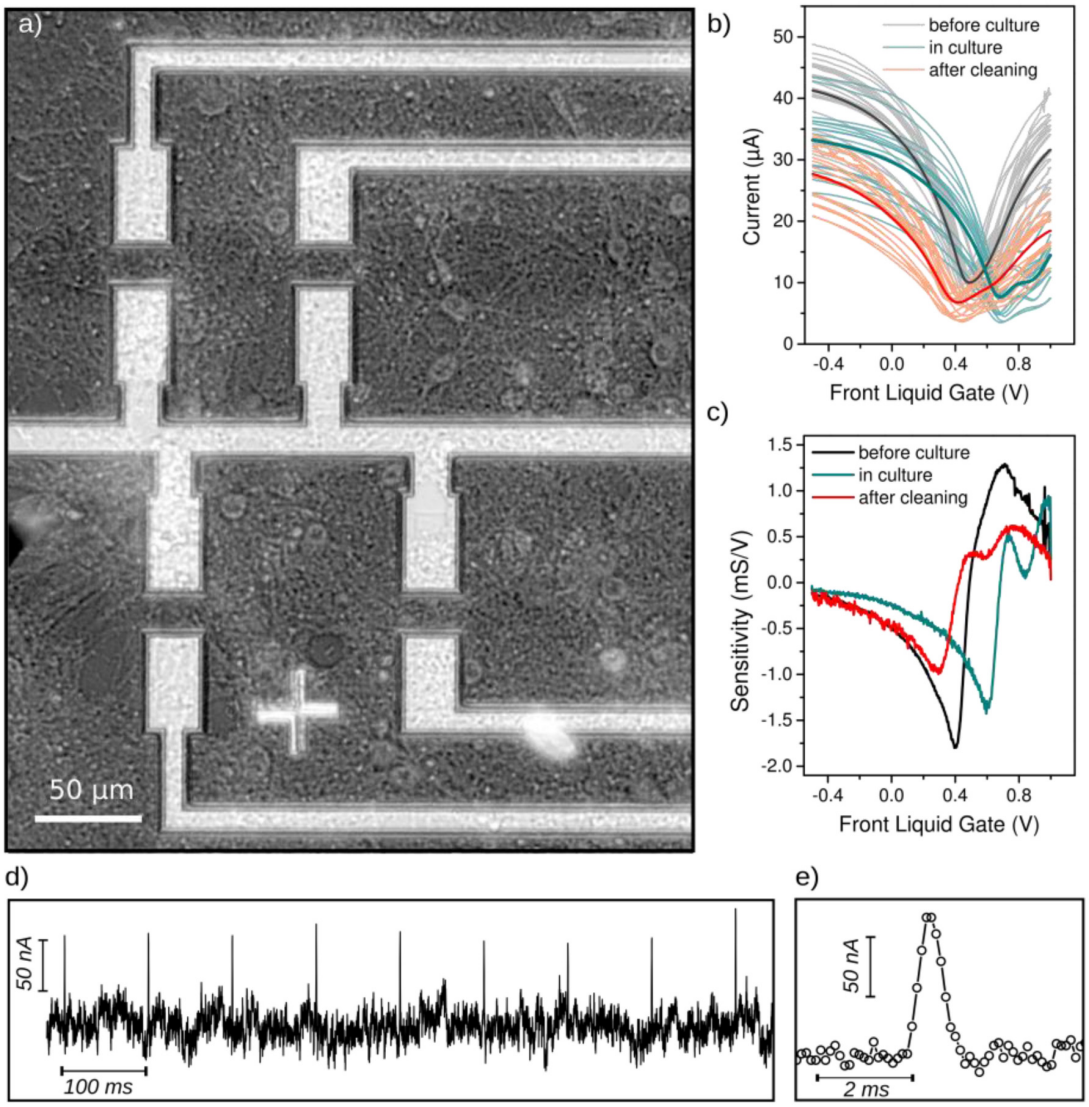
Performance of the G-FETs after 3 weeks of neurons culture. **(a)** Optical micrograph of the dense neuronal networks cultured on G-FETs during 21 days. **(b)** Field effect of liquid gated G-FETs before culture (gray curves), covered with 21DIV neurons (blue curves), and after cleaning the chip (orange curves). Curves represent one device, except the darker one which is the average field effect curves for each condition. All measurements were performed in cell culture medium at V_SD_ = 50 mV. **(c)** Average sensitivity of G-FETs before the culture (black), covered with neurons (blue) and after cleaning (red). The sensitivity was calculated from the average field effect curves in **(b)**. **(d)** Potential pulse detection using G-FETs covered with neurons. A 1 ms long potential pulse V_*p*_ = 500 μV was applied to the cell culture medium through a Pt-electrode; the transistor working point was set to the highest sensitivity, and the current through the FET-channel was measured at V_SD_ = 100 mV. **(e)** Zoomed view of an individual current pulse detected in **(d)**. A Pt-reference electrode was used as the gate electrode for all the measurements.

When the field effect curve is measured with live neurons on top of the FET devices (Figure 5a), a 0.2 V positive shift of the charge neutrality point can be observed along with a general conductance depression (Figure 5b). Also the field effect curve exhibits two local minima suggesting the coexistence of graphene with two well distinct doping. These observations can be clearly attributed to the presence of dense neuron networks adsorbed on the FETs surface, since after cleaning the device, the Dirac point shifts back to less positive gate voltage values, and the double conductance minimum almost disapears. A control experiement was performed without neuron, after 3 weeks of incubation in culture medium at 37°C (Figure S1). This shift of the Dirac point in presence of neurons can be attributed to the negative resting membrane potential (Figure 5a). Also, the excitability of neurons could slightly gates the GFET channel, the current being slightly lower when neurons activity is blocked (~95% of *I*_*DS*_, by adding tetrodotoxin at 0.5 μM in the cells medium) or when neurons are fixed (37% of *I*_*DS*_, after fixing neurons with 4% paraformaldehyde 10 min; Supplementary Figure S2).

After the electrical recordings with neurons, the chip was immersed into warm DI water, resulting in the burst of neurons due to osmotic pressure. The chip was then rinsed several times with DI water and dried. After this simple cleaning procedure, the transfer characteristics of G-FETs were measured again in cell culture medium. After removing the neurons, the Dirac point shifted back to less positive voltage values (being closer to the value before the cell culture). However, the sensitivity of G-FETs was reduced to 50% of the initial value after the cleaning (Figure 5c). To re-use the chips for several cultures, the cleaning process has to be improved. First, DI water should not be used for removing the neurons, since the surface tension of water can lead to partial delamination of the graphene layer. For instance, a cleaning with an enzymatic bath (that does not damage the graphene monolayer) could be used to remove cellular matrix residuals. Also, characterizing the electrodes material with Raman microscopy could provide new insights on the structure of the graphene FETs channel after the cell culture.

The sensitivity of G-FETs is reduced by 25% during the culture (Figure 5c), which might be due to the damaged graphene surface induced by the tension of growing neurons and resulting in a lower mobility. On the other hand, the decreased sensitivity may arise from the reduced field effect due to the presence of a screening neuron layer on the FETs. Indeed, the FETs are still able to detect small potential changes such as, stimulated spike, as shown in Figure 5d, even with a reduced sensitivity. The positive and biphasic change of the drain source current (Figure 5e) strongly resembles the expected shape of evoked neuronal spike. A direct gating by the liquid should rather induce negative and monophasic variation of *I*_*DS*_ such as, shown in Figure 3b without neurons.

This stable performance of G-FETs in the biological environment combined with the excitability of cultured neurons makes the detection of the neuronal activity and particularly single action potentials, using flexible and transparent G-FET feasible.

Nevertheless, further improvements of the devices performance should be investigated for reliable spike tracking and high detection efficiency, in term of number of spike detected per neurons and per devices. Electronics noise could be reduced using suspended graphene or by reducing the density of charges trapped at the interface with the substrate (Cheng et al., 2013). Also higher mobility could be obtained by increasing the crystalline quality of CVD grown graphene, for instance by enlarging the single grain size which should increase the sensitivity of the devices (Gao et al., 2012).

The use of 2D materials could be promising for bio-sensing applications and for interfacing neuronal networks in slices or *in vivo* nervous systems (CNS and PNS), regarding the possibility to combine a transparent digital interface with optical techniques (Kuzum et al., 2014). Also, the use of soft and flexible substrates could (be used to) reduce the inflammatory response and enable drugs delivery, to support the scarring process and the bioacceptance of the artificial interface (Minev et al., 2015; Lee et al., 2016). In addition with the chemical inertness and anti-corrosive properties of graphene, such bio-suitable features are crucial for implementing active scaffolds and long lasting neuronal interfaces in neurosciences and medicine.

## Conclusion

The hippocampal neurons cultured on G-FETs exhibit healthy morphology and spontaneous electrical activity after 19–21 days in culture. The 1–2 h long electrical recordings performed on neurons using G-FETs do not damage the cells, at least no significant change is observed with immuno-fluorescent imaging of neurons after the measurements. Both shape and proteins expressions are as expected for 21 days old cultured neurons. The performance of G-FETs degrades slightly during the culture. However, the FETs are still able to detect small potential pulses on the range of action potential spikes generated by neurons. Both the neuron culturing technique used in this work and the FET performance are sufficient to pave the road for the realization of a cell-FET hybrid device and for recording electrical activity of single neurons with flexible and transparent substrates, useful for the large scale integration of suitable devices for bioelectronics and neuroprosthetics.

## Author contributions

FV, VB, and CD conceived and designed the project. FV and CD performed the devices and the neurons experiments. AB and CD performed the cells culture. ZH, DK, and VB performed the graphene growth. FV and CD analyzed the data and wrote the manuscript with VB and AB.

### Conflict of interest statement

The authors declare that the research was conducted in the absence of any commercial or financial relationships that could be construed as a potential conflict of interest.
