# Supplementary material for: Recording Spikes Activity in Cultured Hippocampal Neurons Using Flexible or Transparent Graphene Transistors
Source: Front Neurosci. 2017 Aug 28;11:466. doi: 10.3389/fnins.2017.00466 (PMC5581354; doi:10.3389/fnins.2017.00466)
Supplement: Supplementary file 1 [file Presentation1.PDF]

Supplementary Material

Recording spikes activity in cultured hippocampal neurons using flexible or transparent graphene transistors

Farida Veliev†, Zheng Han†, Dipankar Kalita†, Anne Briançon-Marjollet‡, Vincent Bouchiat† Cécile Delacour†*

*** Correspondence:** Corresponding Author: cecile.delacour@neel.cnrs.fr

Supplementary Figures

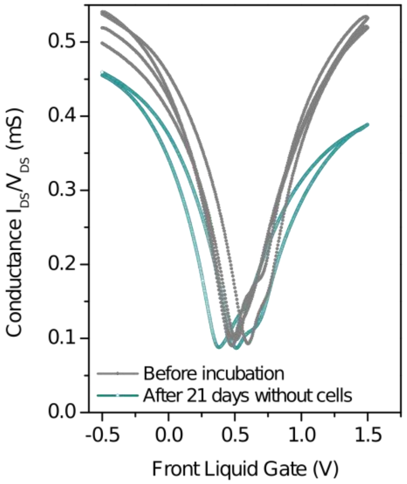

Supplementary Figure S1. Performance of G-FETs after 3 weeks in culture medium at 37°C without neurons. Field effect characteristics of liquid-gated G-FETs (40×50 μm²) on SiO2. A Pt-reference electrode was used as the gate electrode.

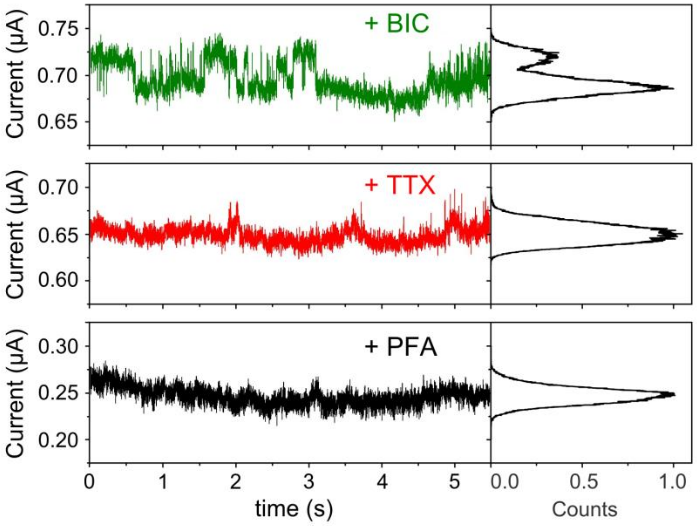

Supplementary Figure S2. Typical effects of tetrodotoxin (TTX) and paraformaldehyde (PFA) on the current traces (left) and corresponding histograms (right) of G-FETs interfaced to cultured neurons (DIV19). The current traces are subsequently recorded at a constant bias voltage VSD = 50 mV and liquid gate potential VG = 0.15 V. A Pt-reference electrode was used as the gate electrode.
